# Improved Characteristics of CdSe/CdS/ZnS Core-Shell Quantum Dots Using an Oleylamine-Modified Process

**DOI:** 10.3390/nano12060909

**Published:** 2022-03-09

**Authors:** Kai-Ping Chang, Yu-Cheng Yeh, Chung-Jui Wu, Chao-Chun Yen, Dong-Sing Wuu

**Affiliations:** 1 Department of Materials Science and Engineering, National Chung Hsing University, Taichung 40227, Taiwan; a3322123@gmail.com (K.-P.C.); larry19890912@livemail.tw (Y.-C.Y.); q0952488342@gmail.com (C.-J.W.); murdocdepp@dragon.nchu.edu.tw (C.-C.Y.); 2 Innovation and Development Center of Sustainable Agriculture, National Chung Hsing University, Taichung 40227, Taiwan; 3 Department of Applied Materials and Optoelectronic Engineering, National Chi Nan University, Nantou 54561, Taiwan

**Keywords:** application of nanomaterials, quantum dots, ZnO, one-pot synthesis, oleylamine, aging test

## Abstract

CdSe/CdS with ZnS/ZnO shell quantum dots (QDs) are synthesized by a one-pot method with various oleylamine (OLA) contents. The crystal structures of the QDs were analyzed by X-ray diffractometry, which showed ZnS diffraction peaks. It was represented that the ZnS shell was formed on the surface of the CdSe/CdS core. Interestingly, QDs with a high OLA concentration exhibit diffraction peaks of ZnS/ZnO. As a result, the thermal stability of QDs with ZnS/ZnO shells exhibits better performance than those with ZnS shells. In addition, the photoluminescence intensity of QDs with ZnS/ZnO shells shows a relatively slow decay of 7.1% compared with ZnS shells at 85 °C/85% relative humidity aging test for 500 h. These indicate that QDs with different OLA modifications can form ZnS/ZnO shells and have good stability in a harsh environment. The emission wavelength of QDs can be tuned from 505 to 610 nm, suitable for micro-LED display applications.

## 1. Introduction

For nearly 2 decades, quantum dots (QDs) have attracted attention and are widely used in sensors, detectors, and solar cells [1,2,3]; they exhibit a broad application prospect in the field of analysis and detection because the quantum confinement effects are accompanied by tunable and excellent fluorescence characteristics such as high fluorescence quantum yield, stable luminescence, wide excitation spectrum, and narrow emission spectrum [4,5]. However, the luminous efficiency of QDs degrades in a harsh environment, including water, thermal heating, and ultraviolet (UV) exposure [6], because of the uneven surface or lack of surfactant of QDs. Therefore, the concept of core–shell heterostructure interface on QDs was proposed, which adopts elements in the same group of the periodic table to remedy defects on the surface of QDs. This concept can effectively improve separation efficiency and suppress charge recombination. These materials of heterojunction in QDs are divided into two types. In type I heterostructures, the core’s conduction and valence band minimums are lower than those of the shell [7,8,9]. An electron can be excited from the valance to the conduction band and will be confined to the inner material. In type II, the core’s conduction band is higher than that of the shell, where an electron will excite from the core’s valance band to the shell’s conduction band [10,11]. However, QD surfaces are very sensitive to their immediate environment [12], which may limit the type II adoption in display applications. To develop high quality and high stability QDs, the type I core–shell structure of CdSe/CdS@ZnS or Zinc oxide (ZnO) QDs has been adopted in various studies, such as light-emitting diodes and biomedical applications. [13,14,15]. The ZnO shell is an ideal candidate as the shell on CdSe/CdS QDs and is usually deposited by atomic layer deposition. However, the photoluminescence (PL) of the QDs may decay because of the interaction of the outer ZnS shell of the QDs during trimethylaluminum pretreatment [16].

On the other hand, in terms of wavelength modulation of CdSe/ZnS QD, the fluorescence emission spectrum of CdSe/ZnS QDs in the visible region can be obtained by changing the size of QDs or modifying the Cd to Zn ratio [17,18], which can be combined with the blue or UV micro-light-emitting diode (micro-LED) backlight to achieve a full-color or monochrome/area color for micro-LED display applications. Traditionally, CdSe/ZnS core–shell QDs can be synthesized by a two-step synthesis method. The first step involves synthesizing and purifying the CdSe/CdS, and the second step entails synthesizing and purifying the inorganic ZnS shell. Although it is easy to control QD size in this synthesis method, it is a costly and time-consuming process because of the complicated calculation of the lattice constant between the concentration of CdSe and ZnS. Hines et al. [19] devised a one-pot method to synthesize CdSe core QDs using dimethylcadmium and tri-n-octylphosphine (TOP)/Se mixed liquor as the precursor of Cd/Se at a high-temperature reaction process. Then, the CdSe/ZnS core–shell is formed by injecting Zn/S/TOP solution. Cho et al. [20] have demonstrated CdSe/CdSe_x_S_1−x_/ZnSe_y_S_1−y_/ZnS core–shell gradient alloy QDs by a one-pot approach and tuned the size of QDs at different reaction times. However, there is a limit on the wavelength of the QDs attained by adjusting the reaction time to control the size of QDs and bandgap because of the interdiffusion process from the shell into the core, increasing the energy bandgap of band-edge luminescence and contraction of the effective size of the CdSe core [20]. Therefore, it is relevant to study the method of synthesizing wavelength-tunable QDs using trioctyl-phosphine (TOP) [21], 1-octadecene (ODE) [22], oleic acid (OA) [23], and oleylamine (OLA) [24] for superior performance and low cost. OLA is a high boiling point polar substance, which can effectively cover the nanometer particles and maintain the stability of the particles; therefore, it is widely used as a surfactant in the preparation of various QDs [25]. However, the effect of OLA concentration on the growth of CdSe/CdS/ZnS core–shell QDs has not been fully elucidated [26].

In this study, we synthesis quaternary CdSe/CdS/ZnS QDs using a one-pot method. Specifically, four different OLA contents are used to adjust the emission wavelength, morphology and growing size of the QDs. The composition of QDs with different OLA contents is analyzed using transmission electron microscopy (TEM), energy-dispersive X-ray spectroscopy (EDS), X-ray photoelectron spectroscopy (XPS), and X-ray diffraction (XRD). In addition, the CdSe/CdS/ZnS/ZnO QDs are synthesized using high concentration OLA. The ZnO shell can also enhance the photostability of QDs [27]. By adopting the OLA in the synthesis, not only can the wavelength of QDs be adjusted but the formation of the shell can also be optimized. Furthermore, the PL intensity (PLI) decay of QDs with different OLA contents at high-temperature and high-humidity durability is analyzed.

## 2. Materials and Methods

### 2.1. Materials

Zinc acetate [Zn(OAc)2, 99.99%], cadmium oxide (CdO, 99.99%), selenium (Se, 99.99%), sulfur (S, 99.99%), TOP (90%), ODE (90%), OA (90%), OLA (80–90%), ethanol, and anhydrous toluene were purchased from Sigma-Aldrich (Uni-onward Trade Co., Ltd., New Taipei City, Taiwan) and used without any further purification [TOP:3(mL), CdO:25.682(mg), Zn(act)2:733.9(mg), OA:10.6(mL), ODE:10(mL), Se:15.79(mg), S:128.26(mg)].

### 2.2. Methods

**Figure 1:**
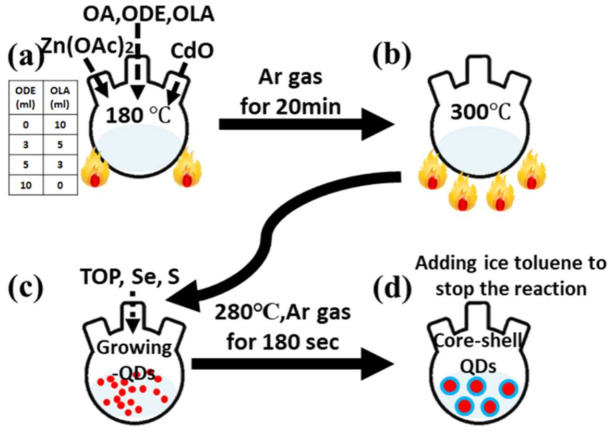
Schematic process flows of QDs. (**a**) The mixture was heated to 180 °C and degassed by Ar for 20 min, (**b**) the temperature was increased to 300 °C, (**c**) the TOP, Se and S were injected into the four-neck flask by using a syringe, and (**d**) rapidly cooled down by adding ice toluene to stop the reaction.

First, TOP, S, and Se were loaded into a bottom using an ultrasonic machine to prepare the mixture. Then, to prepare the precursor, CdO, Zn(OAc)_2_, and OA were loaded into a four-neck flask along with different amounts of OLA (0, 3, 5, and 10 mL) and ODE (0, 5, 7, and 10 mL) (Figure 1a). Then, the mixture was heated at 180 °C until the cloudy solution clarified. Next, the clarified mixture was heated to 300 °C (Figure 1b), and the solution of TOP, S, and Se was injected into the four-neck flask using a syringe (Figure 1c). The temperature was decreased to 280 °C and held for 180 s. The solution was rapidly cooled down by adding ice toluene to stop the reaction (Figure 1d). Finally, the solution was diluted with alcohol and acetone and then centrifuged at 7000 rpm for 10 min to purify the QDs. The photoluminescence quantum yield (PLQY) values for samples with different OLA contents were measured with an absolute PLQY system (HORIBA Jobin Yvon, FluoroMax ^®^-4 System with Integrating Sphere Accessories, Edison, NJ, USA). The samples were loaded into a sphere, which was coated with a reflective surface of barium sulfate-based film to capture all the light going in and out of the sphere. The PLQY can be calculated from the Equation (1) [28]:

(1) $$\mathrm{PL}\;\mathrm{QY}\;=\frac{E_{c}-\left(1-A\right)\;\times \;E_{a}}{L_{a}\times A}=\frac{E_{c}-E_{a}}{L_{a}-L_{c}}$$

where A is the absorbance of the sample at the excitation wavelength, $E_{c}$ is the fluorescence emission area in PL spectrum, $E_{a}$ is the integrated luminescence from the sample caused by indirect luminescence from the sphere, $L_{a}$ is the scatter intensity of a blank, and $L_{c}$ is the scatter intensity area of the sample.

## 3. Results and Discussion

**Figure 2:**
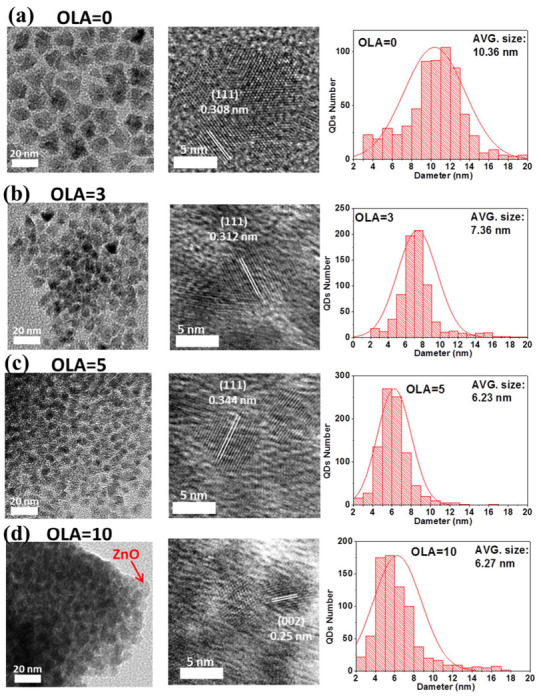
Top-view HRTEM images and the size distributions of QDs with (**a**) OLA = 0, (**b**) OLA = 3 (**c**) OLA = 5, and (**d**) OLA = 10 mL.

The different sizes of CdSe/CdS with ZnS or ZnO shell QDs are prepared by one-pot synthesis with different OLA contents. Figure 2 shows the top-view high-resolution TEM (HRTEM) images of the QDs with 0, 3, 5, and 10 mL OLA content. The diameters of the QDs decrease as the OLA content increase from 0 to 10 mL (Figure 2). The larger diameter of the QDs with OLA = 0 can be attributed to the thicker shell of the QDs and it shows the planar d-spacing of 0.308 nm, which can be indexed as ZnS (111) [29]. The order of elements in composition will follow the formation kinetic rate (CdSe > CdS > ZnSe > ZnS) [12]. In the synthesis process at 180 °C, the precursors of CdO/Zn(OAc)_2_ in OA, OLA, and ODE solution form Zn(OA)_2_, Cd(OA)_2_, and [Zn(OAc)_2_]–OLA complex, respectively.

When the reaction temperature reaches 300 °C, the solutions of TOP, S, and Se are injected into the four-neck flask, and the QDs start to form. An increase in OLA content leads to a decrease in the size of ZnSe/ZnS shell structure because of the higher [Zn(OAc)_2_]–OLA and lower Zn(OA)_2_, which will reduce the probability of reaction between Zn(OA)_2_ and Se^2−^. However, the reaction probability between Cd(OA)_2_, Se^2−^, and S^2−^ will relatively increase because of the abundant Se^2−^ and S^2−^. Hence, the size of the CdSe/CdS core will increase and the size of the shell will decrease. Overall, the diameters of QDs tend to decrease. From Figure 2c,d, as OLA content increases from 5 to 10 mL, significant size changes are seen in the QDs compared to the QDs with OLA = 0. With OLA = 5, the HRTEM image of QDs shows planar d-spacing of 0.344 nm, and it can be indexed as CdSe (002) plane of the core. Due to the shell decreases of the ZnS, the d-spacing value of synthesized QDs is mainly determined by the core of the CdSe. The bandgap of the CdSe (bulk bandgap ≅ 1.7 eV) is also smaller than the CdS (bulk bandgap ≅ 2.53 eV), ZnSe (bulk bandgap ≅ 2.7 eV), and ZnS (bandgap ≅ 3.6 eV). Consequently, it will induce the redshift of the PL emission. With OLA = 10, the HRTEM image of QDs shows planar d-spacing of 0.25 nm, and it can be indexed as ZnO (002) plane of the shell [29]. The core size decreases because the probability of reaction between Zn(OA)_2_ and Se^2−^ is reduced to a minimum. Moreover, the higher [Zn(OAc)_2_]–OLA will form a ZnO shell, which may limit the core size after the CdSe formation. Hence, the ratio of the CdSe in the CdSe/CdS core may increase.

**Figure 3:**
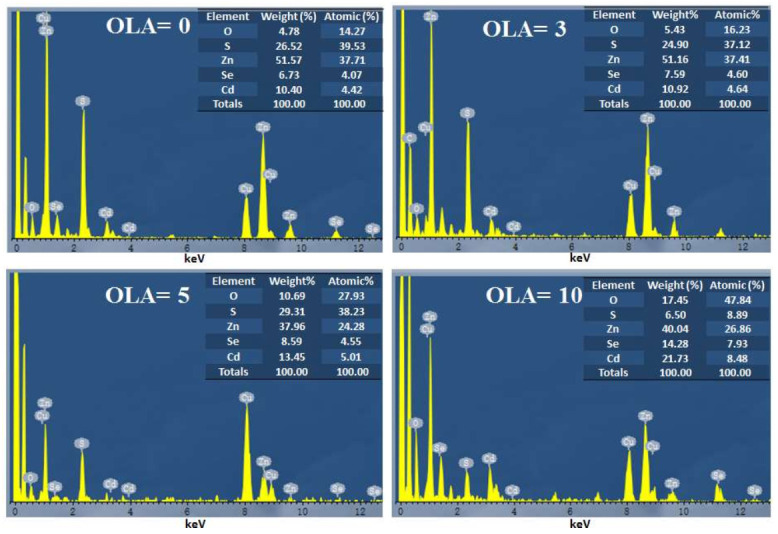
EDS spectra of as-obtained QDs obtained at different OLA.

**Figure 4:**
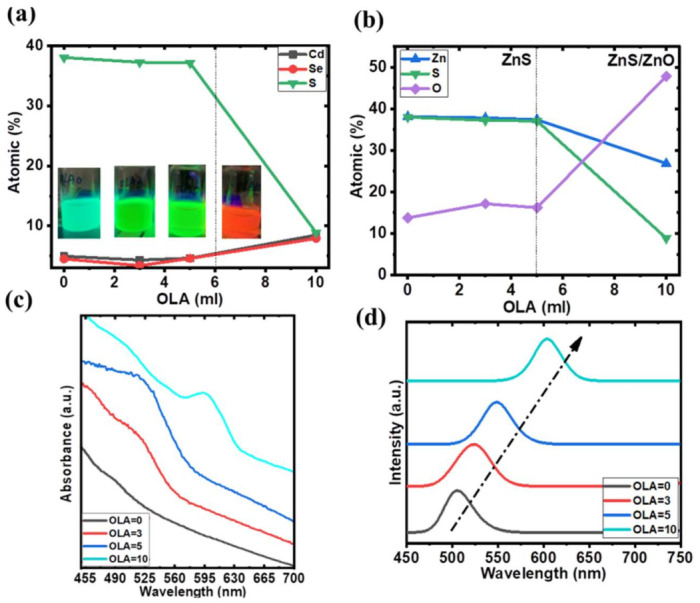
Elemental composition of (**a**) CdS/CdSe core and (**b**) ZnS/ZnO shell QDs obtained at different OLA; (**c**) UV-vis absorption spectra, (**d**) PL spectra of QDs with different OLA content.

Next, the chemical compositions of the core–shell QDs are investigated by energy dispersive X-ray spectroscopy (EDS). As shown in Figure 3, four EDS spectrum analyses from QDs with different OLA revealed different proportions of O, S, Zn, Se, and Cd (%wt). The weight-percent calculations excluded carbon and copper from the copper grid and carbon film on the copper grid background. Figure 4a,b show the chemical compositions of the QDs, which were extracted by EDS spectra. As the OLA content increased from 0 to 5 mL, the atomic percentage of the CdSe/CdS core remained unchanged. This indicates that there is no significant change in the chemical composition of the core. The average diameter of the CdSe/CdS core might increase when the OLA content is adjusted from 0 to 3 mL with an increase in [Zn(OAc)_2_]–OLA, as shown in Figure 2a,b. While increasing the OLA content from 5 to 10 mL, the size of the QDs remains the same and the ZnO shell is observed in QDs-OLA 10, as shown in Figure 2c,d. The EDS spectrum analyses from QDs with OLA = 10 revealed that the atomic percentage of the S in QDs with OLA = 0 is 39.5%. However, the atomic percentage of the S in QDs with OLA = 10 is only 8%. This implies that the concentration of CdS (bandgap ≅ 2.53 eV, theoretical wavelength ≅ 490 nm) and ZnS in QDs structure with OLA = 10 is lower than QDs with OLA = 0.

Figure 4c shows the UV-vis absorption of QDs prepared with different OLA contents. The absorption wavelengths of QDs-OLA 10 exhibited an obvious redshift (from 550 to 597 nm) compared to QDs-OLA 5, which may be attributed to the different compositions of the QDs.

In Figure 4d, the redshift of the emission wavelength is attributable to the large core size caused by the quantum confinement effect, while the OLA content increased from 0 to 3 mL. With a little higher OLA content, the concentrations of Zn(OA)_2_ will also decrease because of the increasing [Zn(OAc)_2_]–OLA. Depending on the kinetic energy, the Zn(OA)_2_ plays the main role in the formation of ZnSe (bulk bandgap ≅ 2.7 eV) and ZnS in the QDs. The ZnSe gets a little thinner, which means the probability of the CdSe core is increased. The formation rate followed the kinetic energy, i.e., CdSe > CdS > ZnSe > ZnS. Hence, in the OLA = 3, the CdSe core may become bigger than the QDs with OLA = 0 and induce the PL redshift. Due to the thinner shell of the QDs with OLA = 3, this also shows a faster PLI degradation than QDs with OLA = 0.

As the OLA content increased from 5 to 10 mL, the composition of S and O dramatically decreased and increased, respectively (Figure 4b). This indicates that the QDs exhibit enormous differences in structure with different OLA content. The PL QY was also estimated to be approximately 42.7%, 35.8%, 23.2%, and 45% for QDs-OLA 0, QDs-OLA 3, QDs-OLA 5, and QDs-OLA 10, respectively. Because ZnO has a lower lattice mismatch with CdSe than ZnS, it can provide strong confinement for the CdSe QD cores and remove the surface defects. [14,23] In addition, the more the content of the OLA, the longer the PL wavelength can be achieved (Figure 4d). The atomic percentage of O drastically increased with the highest OLA content. This indicates that the ZnO shell may be coated on the QDs because of excessive [Zn(OAc)_2_]–OLA.

**Figure 5:**
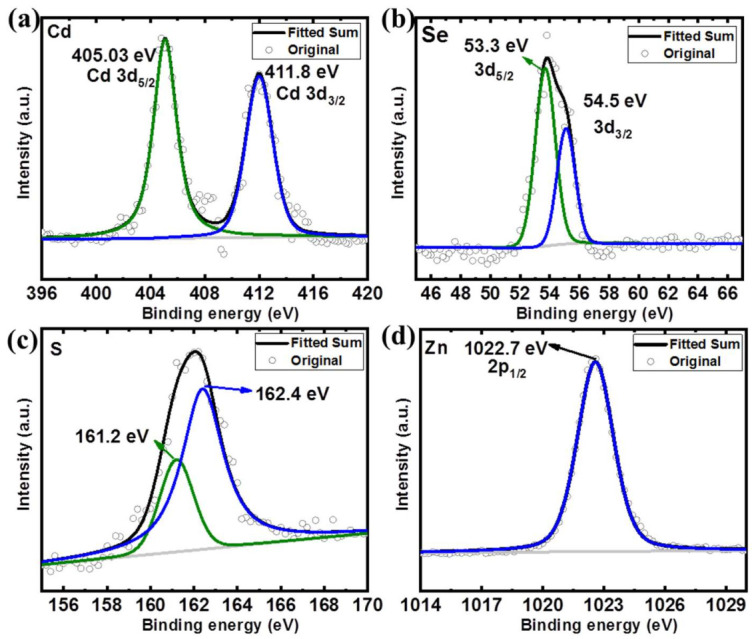
The XPS spectra of (**a**) Cd 3d, (**b**) Se 3d, (**c**) S 2p, and (**d**) Zn 2p in the QDs-OLA 0.

**Figure 6:**
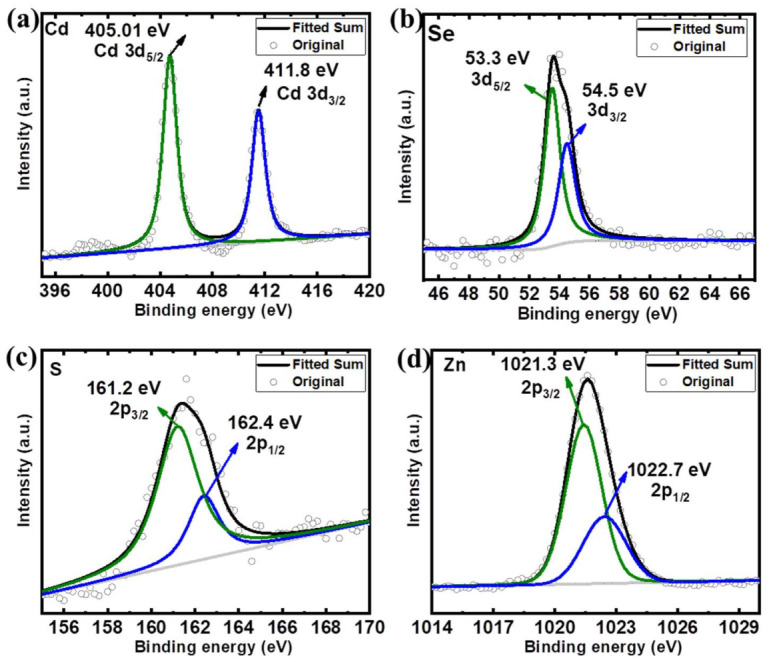
The XPS spectra of (**a**) Cd 3d, (**b**) Se 3d, (**c**) S 2p, and (**d**) Zn 2p in the QDs-OLA 10.

Figure 5 and Figure 6 show the XPS spectra of the QDs-OLA 0 and QDs-OLA 10. In Figure 5a and Figure 6a, two peaks with binding energies of 405 and 411.8 eV can be attributed to Cd 3d [30]. In Figure 5b and Figure 6b, the Se 3d_5/2_ and Se 3d_3/2_ peaks with binding energies of 53.3 and 54.5 eV, respectively, are attributed to the Se^2−^ in CdSe and ZnSe, thus confirming the formation of CdSe and ZnSe [30,31]. In Figure 5c and Figure 6c, the peaks located at 161.2 eV and 162.4 eV can be attributed to the bivalent S^2–^ state and the 161.2 eV can represent the Zn−S bond [32]. In Figure 5d, the peak with binding energy of 1021.3 eV can be attributed to the Zn^2+^ that exists in the ZnSe [33,34]. The binding energy of Zn 2p_3/2_ is approximately at 1022.7 eV, which is close to the reported peak binding energy of ZnS shell in CdSe/ZnS core–shell QDs [35,36]. In Figure 6d, two peaks with binding energies of 1021.3 and 1022.7 eV can be found, which are assigned to Zn^2+^ in the form of ZnO [27] and ZnS [37].

To estimate the thickness of the QDs core, the XPS intensity ratio of the S 2p_1/2_ and Se 3d_5/2_ can be used to estimate the thickness of the CdSe/CdS QDs structure [38,39]. In the XPS spectra of the QDs-OLA 0, the intensity ratio of the S 2p_1/2_/Se 3d_5/2_ is approximately 18.4, which is very close to the 3 nm thick CdS in Florian W. et al. [39]. In the XPS spectra of the QDs-OLA 10, the peaks ratio of S 2p_1/2_/Se 3d_5/2_ is close to 1.26, which can assume that there is little CdS thickness in the core of QDs. Hence, the smaller bandgap of the CdSe (1.7 eV) becomes a dominant component in the core. The bandgap of CdSe (1.7 eV) is lower than the CdS (2.53 eV), which will induce the redshift of the PL emission wavelength. The quantitative analysis of the ZnSe/ZnS/ZnO shell still needs to be further simulated by the SESSA (Simulation of Electron Spectra for Surface Analysis) software and will be realized later on.

**Figure 7:**
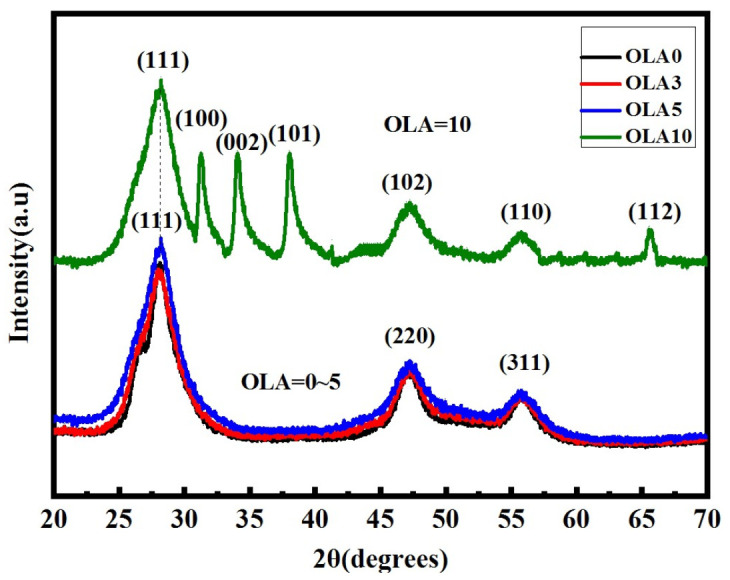
X-ray diffraction (XRD) patterns of core–shell QDs with OLA content.

The XRD patterns are shown in Figure 7. There are three diffraction peaks of QDs with 0–5 mL OLA content located at 2θ values of 28.1°, 47.2°, and 55.8° (JCPDF card no. 05-0566), which indicate that ZnS shell has formed on the CdSe core. In the QDs of 10 mL OLA content, several diffraction peaks located at 2θ values of 31.61°, 34.26°, 36.10°, 47.37°, 56.40°, 62.68°, and 67.72° appeared, corresponding to the (100), (002), (101), (102), (110), (103), and (112) planes, respectively, of the wurtzite ZnO structure (JCPDS card no. 03-065-3411). The asymmetric peaks of the wurtzite structure of ZnO might be attributed to the powder of the QDs [40]. In this work, before the XRD measurement, the QDs solutions will be centrifuged and dried on the bench. Then the QDs will become a powder at the macroscopic level and will be put on the glass for the XRD measurement. Because of the disorder of powder, the peaks of the ZnO might be asymmetric. Another possible reason is that the ZnO is coated on a group of QDs and it might induce the disorder structures.

**Figure 8:**
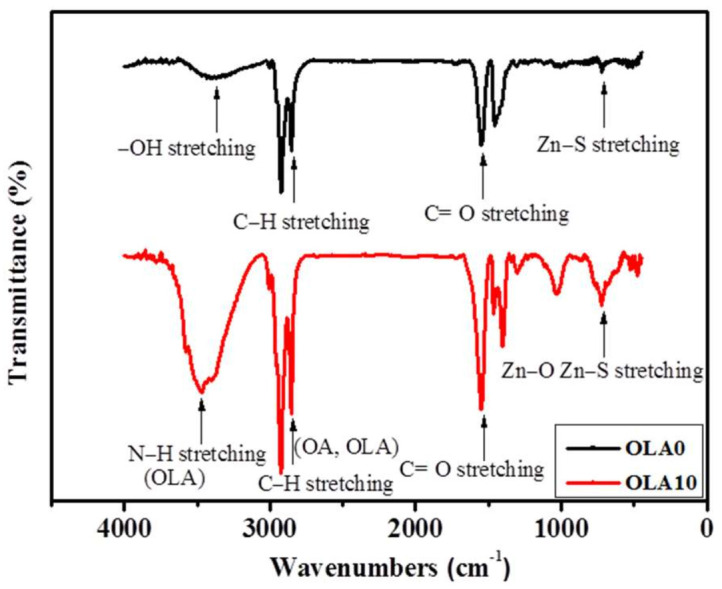
FTIR spectra of QDs with different OLA content (0 and 10 mL).

Figure 8 shows the Fourier-transform infrared (FTIR) spectra of QDs with OLA content of 0 and 10 mL. The signal of FTIR present strong absorption peaks at 2986 to 3686 cm^−1^. This signal is characteristic of the carboxylic acid O–H stretching mode of OA and N–H stretching vibrations of OLA, respectively. The strong absorption peaks located from 2853 to 3005 cm^−1^ are attributed to the =C–H and C–H stretching vibration of OA and OLA, respectively. In the wavenumber region of 1000–1750 cm^−1^, the absorption peak can be ascribed to the OA and OLA. The remaining peaks between 600 and 650 cm^−1^ are attributed to the Zn–O and Zn–S stretches. This implies that OLA could form ZnO shell by [Zn(OA)_2_]–OLA complex, and finally form ZnS/ZnO shell.

**Figure 9:**
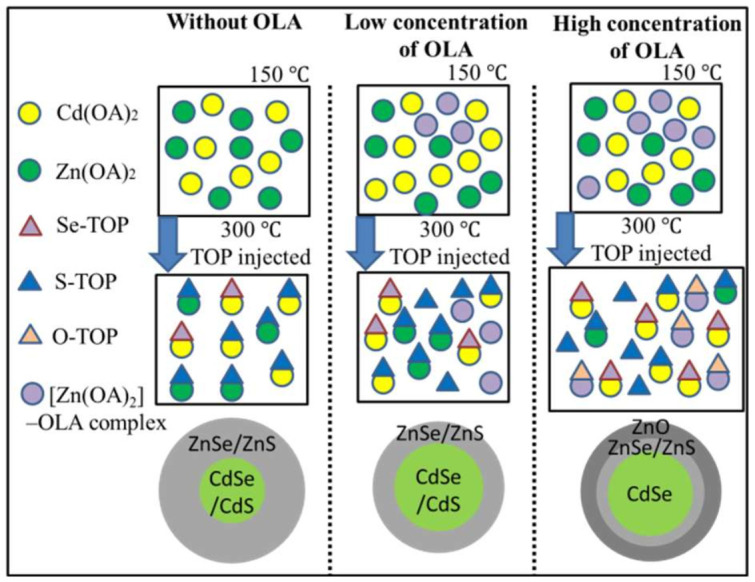
The illustration of the chemical reaction mechanisms by one-pot chemical synthesis of CdSe/CdS/ZnS QDs with different concentration of OLA.

To elucidate the chemical reaction mechanisms, we propose a schematic diagram of the possible reaction mechanisms of a one-pot chemical synthesis method for the preparation of QDs with low/high OLA content (Figure 9). The precursors (CdO and Zn(OAc)_2_) in OA and ODE solutions form Zn(OA)_2_ and Cd(OA)_2_, respectively, when the reaction temperature reaches 150 °C. When the reaction temperature reached 300 °C, Se–TOP and S–TOP solutions were quickly injected into the reaction solution. At this time, the core of CdSe/CdS begins to form. Then, the ZnSe/ZnS is formed on the surface of the core. With a low OLA content, the core size of CdSe/CdS increases with a decrease in the ratio of Zn(OA)_2_. Hence, the concentrations of Se and S increased. With the high OLA content, the concentrations of Zn(OA)_2_ and Se/S reach the minimum and maximum, respectively. Hence, CdSe formed in the beginning and consumed most of the Cd. The main composition of the core is formed by CdSe. Moreover, a high [Zn(OAc)_2_]–OLA will form a ZnO shell on the surface of QDs.

In general, in the QDs structure, the strain effect is existent. The strain resulting from the lattice mismatch between the core and shells may affect the emission energy from QDs. It is present not only at the interfaces but within the entire QDs core, which affects the bandgap. Introducing the middle shell (CdS or ZnSe) sandwiched between CdSe core and ZnS outer shell allows considerable reducing strain inside QDs because CdS and ZnSe have the lattice parameter intermediate to those of CdSe and ZnS [41]. The structure of the QDs-OLA 0 is CdSe/CdS/ZnSe/ZnS. In the XPS spectra of the QDs, the Se 3d_3/2_ peaks with binding energies of 54.5 eV are attributed to the Se^2−^ in ZnSe, thus confirming the formation of ZnSe [32]. However, there is still a compressive strain on the core of the CdSe/CdS [42], and the energy gap increases with applied compressive force and decreases under tensile strain [42,43]. Therefore, the strain will induce the blueshift of the emission wavelength and it is consistent with the PL spectra. In the QDs-OLA 10, the structure is CdSe/ZnSe/ZnS/ZnO. In comparison with the CdSe/CdS core, the smaller bandgap of the CdSe core in the QDs-OLA 10 will induce the redshift of the PL spectra. Furthermore, in the simulation of the CdSe/ZnSe core-shell nanocrystals [44], there is a tensile strain on the CdSe core. The stress caused by the lattice mismatch between ZnO and ZnS will also induce the redshift of the emission wavelength [45]. Hence, in the QDs-OLA 10, the strain effects might lead to the smaller bandgap of the CdSe and contribute to the redshift in the PL spectra.

**Figure 10:**
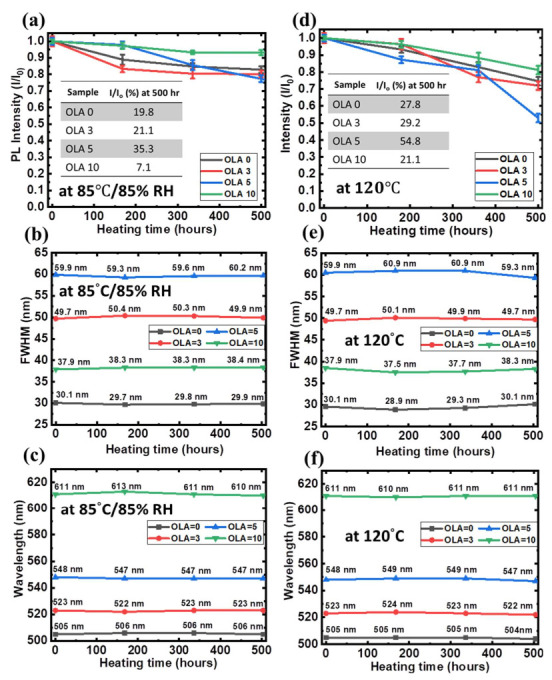
Thermal stability tests of QDs on glass at 85 °C/85% RH and 120 °C (**a**,**d**); traces of PLI, (**b**,**e**) FWHM, and (**c**,**f**) wavelength during 500 h.

Figure 10a shows the aging tests of QDs at 85 °C/85% relative humidity (RH) for 500 h. The QDs with 10 mL OLA content can effectively decelerate the fluorescence decay rate (7.1% PLI decay at 500 h) because of the ZnS and ZnO shell coating on the surface of the QDs core. In the QDs with 5 mL OLA content, the rapid degradation of PLI and the wide full width at half maximum (FWHM) of the peaks are observed (Figure 10b). This can be attributed to the fact that the thickness of the shell is too thin to protect the QD core. The aggregation of the QDs may also significantly increase because of the wider FWHM. The dependence of QDs thermal stability at 120 °C on the heating time is also studied (Figure 10d). It should be noted that the PLI decay rate in the QDs with 10 mL OLA content is slower (21.1%) than the others. The properties of core–shell QDs with ZnS/ZnO shell can be further improved, such as by having strong PLI and thermal stability. In Figure 10b,e, the traces of wavelength in all cases are stable after 85 °C/85% RH or 120 °C for 500 h.

## 4. Conclusions

We illustrated that the CdSe/CdS/ZnS core–shell QDs with emission wavelengths from 505 to 610 nm can be realized for the first time using a simple one-pot synthesis in the presence of OLA contents. The CdSe/CdS core and ZnS/ZnO shell of the QDs were examined by HRTEM. Furthermore, based on XRD, FTIR, XPS, and EDS analyses, the chemical structure of QDs is demonstrated and the ZnO shell is formed on the surface of QDs with a high concentration OLA. An increase in OLA content leads to a decrease of the atomic percentage of S in the CdSe/CdS core, which shows the redshift of the PL emission spectra. The strain resulting from the lattice mismatch between the core and shells may affect the emission energy from QDs. We have also investigated the PLI and QY of QDs by the absolute PLQY system instead of standard organic dye. During the measurement of optical performance at 85 °C/85% relative humidity, the QDs with ZnO shell show superior thermal and humidity stability with a lower than 7.1% PLI decay rate and ±2 nm wavelength shift. These results demonstrated that the wavelengths of QDs can be customized and closer to red and green light, which can combine with the blue or UV micro-LED backlight for long-term operation and achieve a full-color or monochrome/area color for future micro-LED display applications.

## Data Availability

The data that support the findings of this study are available from the corresponding authors upon reasonable request.
